# Both the extended neck position and insertion of a supraglottic airway device increases the height of the cricothyroid membrane in females. A prospective observational study^☆^

**DOI:** 10.1016/j.bjao.2024.100364

**Published:** 2024-12-20

**Authors:** Ankita Miglani, Sandeep Miglani, Hassan M. Tawfik, Thomas Drew

**Affiliations:** 1 The Rotunda Hospital, Dublin, Ireland; 2 Portiuncula Hospital, Galway, Ireland; 3 Beaumont Hospital, Dublin, Ireland; 4 RCSI University of Medicine and Health Sciences, Dublin, Ireland

**Keywords:** cricothyroid membrane, cricothyroidotomy, Difficult Airway Society guidelines, eFONA, i-gel®, supraglottic airway device

## Abstract

**Background:**

Emergency Front of Neck access **(**eFONA) via cricothyroidotomy using a size 6 internal diameter tracheal tube is recommended by the Difficult Airway Society in the event of a ‘can't intubate, can't oxygenate’ (CICO) scenario in adults. There is a lack of clear guidance on whether to retain or remove a previously inserted supraglottic airway device (SAD) before eFONA. We aimed to study the effect of both neck extension and insertion of an SAD on sagittal cricothyroid membrane (CTM) height.

**Methods:**

We recruited 40 adult female patients attending for minor gynaecological surgery under general anaesthesia and suitable for an SAD. Sagittal ultrasound images of the CTM were obtained in the neutral and extended neck position, both before and after insertion of the i-gel® (160 images). The CTM height was measured from the images by a blinded assessor and the data analysed to determine the magnitude of change in CTM height and its relevance for cricothyroidotomy.

**Results:**

There was a significant difference in the height of the CTM between the groups (*P*<0.001). The extended neck position accounted for 10% increase over the neutral position. Inserting an i-gel® and extending the neck increased the CTM height by 26% over neutral position, thereby lengthening it sufficiently to accommodate a size 6.0 tracheal tube in 100% of the patients.

**Conclusions:**

Both neck extension and the insertion of an i-gel® increased the sagittal height of the CTM. This suggests there may be benefit to retaining or re-inserting an SAD during eFONA.

Emergency Front of neck access (eFONA) is the final step in management algorithms for a ‘can't intubate, can't oxygenate’ (CICO) scenario in adults.^1^ CICO scenarios are rare but account for >25% of all anaesthesia-related deaths.^2^ Supraglottic airway devices (SADs) are recommended as ‘plan B’ to provide a route for oxygenation if tracheal intubation fails. If there is failure to oxygenate using an SAD during a CICO situation, it is currently recommended to remove the SAD for an attempt at facemask ventilation before proceeding to eFONA via cricothyroidotomy.^1^ The Difficult Airway Society recommends a 6.0 mm internal diameter tracheal tube to be placed as part of a ‘scalpel–bougie–tube’ technique during eFONA.^1^ It is recommended to continue to provide oxygen 100% during eFONA using an SAD, a tightly fitting face mask or nasal insufflation^1^ but the advantage of one technique over another has not been established. Results of the fourth National Audit Project showed that although cricothyroidotomy was the rescue emergency surgical airway technique of choice for anaesthetists, a failure rate of ∼65% was observed.^3^

Differences in cricothyroid membrane (CTM) dimensions have been demonstrated among population groups by age, sex, and height, by studies using ultrasonography in healthy volunteers.^4^ Attempting to pass a tracheal tube that is too large through the CTM may result in failure to oxygenate and injury to the laryngeal cartilages or adjacent airway structures.^5^ Localisation of the CTM has been demonstrated to be more difficult in females irrespective of body habitus.^6^ This study aimed to compare the changes in anatomical proportions of the CTM with neck extension, both before and after insertion of an SAD device in females.

## Methods

**Fig 1:**
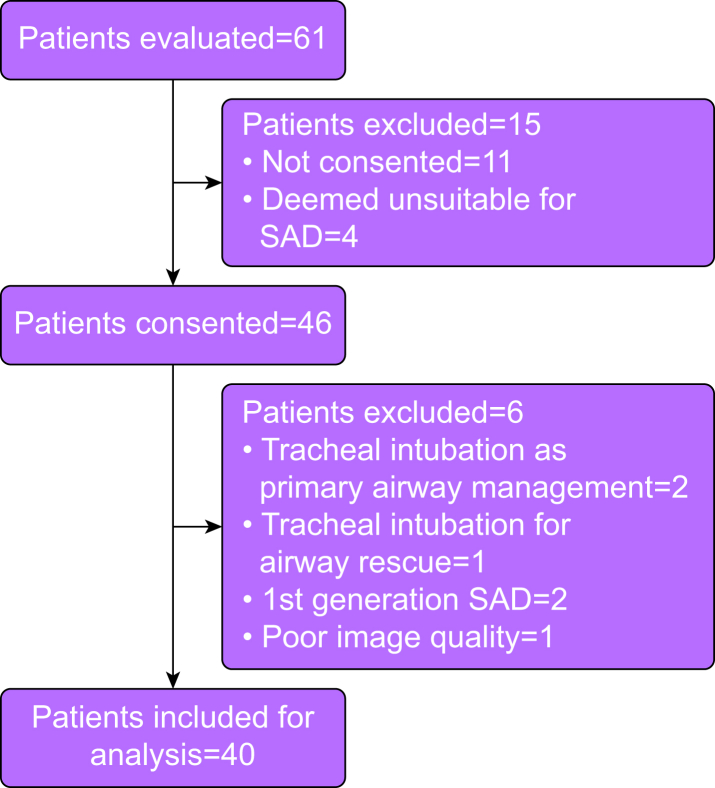
Flow diagram of participants enrolled in the study. SAD, supraglottic airway device.

This prospective observational study was conducted after approval by the research and ethics committee of The Rotunda Hospital, Dublin, Ireland, (REC-2021-026, 15 February 2022) which is a university-affiliated tertiary level centre for obstetrics and gynaecology. Written informed consent was obtained from female patients presenting for elective minor gynaecological surgery. Recruitment took place from April to June 2022 and the project was conducted in accordance with the Strengthening and Reporting of Observational Studies in Epidemiology guidelines. Patients had been adequately fasting for general anaesthesia and had been deemed suitable for an SAD for airway management by the anaesthetist responsible for the clinical care of the patient. The SAD selected for use in this study was the size 4 i-gel® (Intersurgical, Wokingham, UK). Exclusion criteria included age <18 yr or >65 yr, weight <50 kg, previous neck surgery or radiotherapy, inability to lie supine, unsuitability for using a supraglottic device, and inability to give informed consent (see Fig. 1).

**Fig 2:**
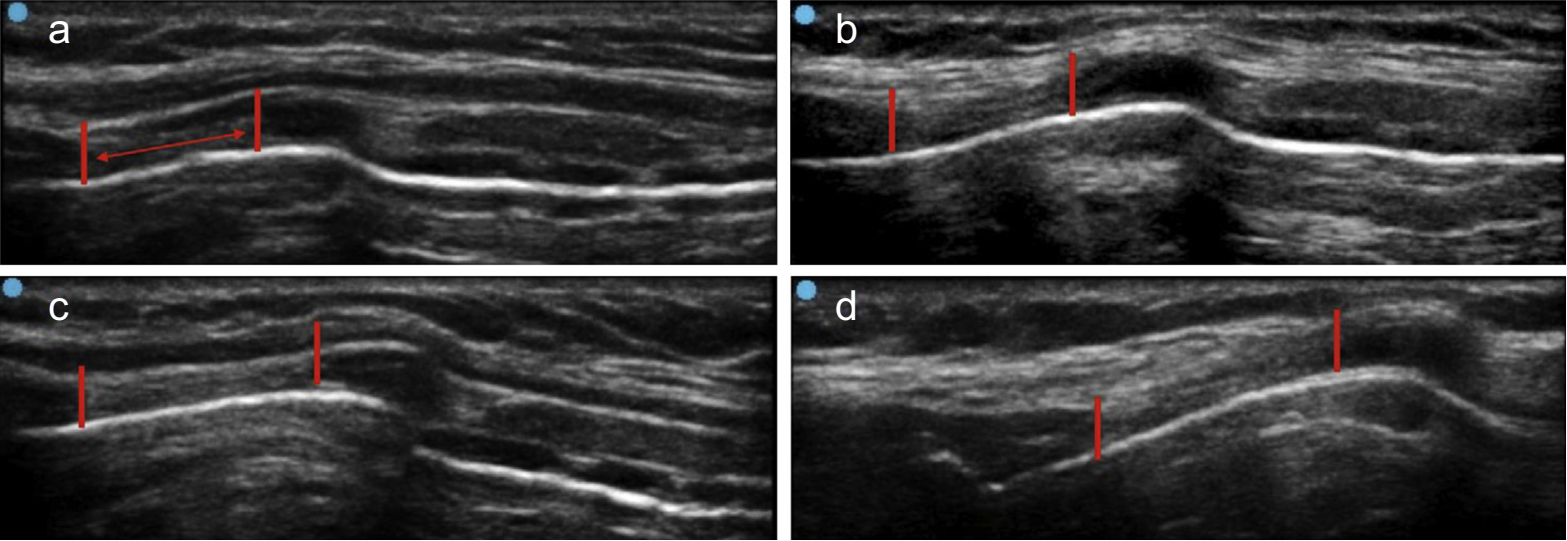
Four sequential front of neck longitudinal ultrasound images from a study participant showing change in CTM height. (a) Neutral neck position, no SAD; (b) extended neck position, no SAD; (c) neutral neck position, SAD *in situ*; (d) extended neck position, SAD *in situ*. Red markings indicate the superior and inferior extent of CTM. Note the cricoid cartilage pushed anteriorly. CTM, cricothyroid membrane; SAD, supraglottic airway device. (For interpretation of the references to colour in this figure legend, the reader is referred to the Web version of this article.)

On arrival to the operating theatre, standard anaesthesia monitoring and preparation were performed. A portable ultrasound machine (Sonosite Micromax™, WA, USA) fitted with a soft tissue longitudinal/linear probe (L38e/10-5 MHz) was used to study the CTM in each patient. Four ultrasound images were captured and analysed for each patient as follows. An ultrasound scan of the front of the neck was performed using the systematic approach described by Kristensen,^7^ and the CTM was identified in the sagittal plane, with the patient's head in a neutral position. The image was captured and stored (Fig. 2a). Patients were then asked to extend the neck as much as comfortably possible and the ultrasound scan of the front of the neck was repeated and the image captured (Fig. 2b). After induction of general anaesthesia, an i-gel® was inserted by the anaesthetist responsible for the patient's care. Once the airway device was secured, ultrasound images of the front of the neck were again captured in the neutral (Fig. 2c) and extended positions (Fig. 2d). After the study recruitment was complete, 160 images were anonymised, downloaded, and randomised. The height of the CTM was measured as the distance between the cranial border of the cricoid cartilage and the caudal border of the thyroid cartilage.

Digital measurements were performed using PixelStick (Plum Amazing Software, Princeville, HI, USA). Data were analysed and figures were produced using Prism (version 7; GraphPad, La Jolla, CA, USA). Continuous data are presented as mean (standard deviation [sd]) where appropriate and 95% confidence intervals (CI) for mean differences are presented. Normality testing was performed for continuous data by assessing skewness and kurtosis of data and using the Shapiro–Wilk test. Subsequently, the significance for continuous data was determined using one-way repeated measures analysis of variance.

The mean height of the CTM in females is ∼10 mm.^8^^,^^9^ To detect a difference of 12% in the height of the CTM between the groups a sample size of 148 measurements, 37 patients with four images each, is required. A sample size of 40 women was determined in total before the study commenced, to allow for three patients where measurements might have been difficult to obtain, or the study aborted to facilitate safe conduct of the anaesthetic.

## Results

We approached 46 patients for consent, of whom 40 were included in the final analysis (Fig. 1). The patient characteristics are shown in Table 1. The study population showed wide variability in characteristics which is likely to represent the variation encountered in clinical practice (Table 1).

**Table 1:** Patient characteristics. Only female patients were recruited. sd, standard deviation.

	Mean	sd	Range
Age (yr)	46	12	19–65
BMI	26.1	4.5	17.9–39.5
Height (cm)	163	7	152–176
Neck circumference (cm)	34.5	2.7	28.5–40
Thyromental distance (cm)	8.8	1.4	6–12
Sternomental distance (cm)	17.3	1.5	13–21

**Fig 3:**
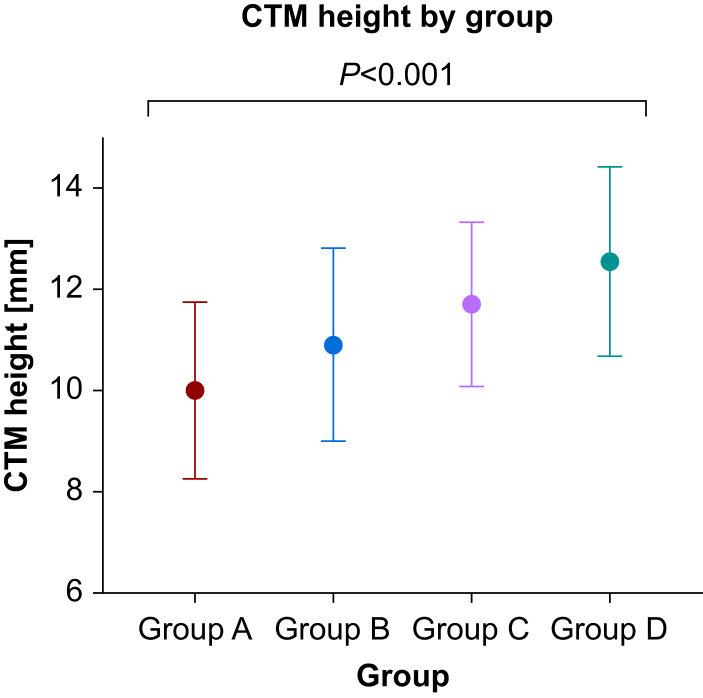
Mean (standard deviation) CTM height by group. Group A: no SAD—neutral position, Group B: no SAD—extended position, Group C: no SAD—neutral position, Group D: no SAD—extended position. CTM, cricothyroid membrane; SAD, supraglottic airway device.

There was a significant difference in the height of the CTM between the groups (*P*<0.001) (Fig 2, Fig 3). The mean (sd) height of the CTM was 9.9 mm (1.7 mm) in Group A, 10.9 mm (1.9 mm) in Group B, 11.6 mm (1.6 mm) in Group C, and 12.5 mm (1.8 mm) in Group D. The extended neck position accounted for a mean of 1 mm (10%) (95% CI: 0.6–1.2 mm) increase over neutral position without an SAD *in situ*. Inserting an SAD increased the height of the CTM by 1.7 mm (17%) (95% CI: 1.2–2.2 mm) in the neutral position and 1.9 mm (95% CI 1.2–2.1 mm) in the extended neck position. When the neck was extended and the SAD was inserted, the height of the CTM increased by 2.6 mm (26%) (95% CI: 2.0–3.1 mm) compared with the neutral position with no SAD *in situ*. The size 6.0 tracheal tube available in our institute (Covidien Shiley™ Hi-Contour Oral/Nasal cuffed tracheal tube, Mansfield, MA, USA) has an exterior diameter of 8.2 mm, 80% of Group A would accommodate a size 6.0 tracheal tube, 92.5% in Group B, 100% in Group C, and 100% in Group D (Table 2).

**Table 2:** Comparative summary of CTM height measurement in the study groups. Mean (sd) measurement of the CTM (mm). CTM, cricothyroid membrane; ET, tracheal tube; SAD, supraglottic airway device; sd, standard deviation.

	Group A (neck neutral, no SAD)	Group B (neck extended, no SAD)	Group C (neck neutral, SAD)	Group D (neck extended, SAD)
Mean (sd)	9.9 (1.7)	10.9 (1.9)	11.6 (1.6)	12.5 (1.8)
% Increase in height	*N*	*N* + 10	*N* + 17	*N* + 26
Proportion accommodating a 6.0 mm ET external diameter (8.2 mm) (%)	80	92.5	100	100

## Discussion

The data from our study demonstrate that in 20% of the participants, the CTM height would not be sufficient to accommodate a size 6 tracheal tube in the neutral neck position. The CTM height increased to 26% more than in the neutral position when the neck was maximally extended with an i-gel® *in situ*. This increase in the height of the CTM was sufficient to accommodate a size 6 tracheal tube in 100% of subjects.

Difficult Airway Society guidelines for rescuing a CICO scenario recommend inserting a lubricated size 6.0 mm internal diameter cuffed tracheal tube through an incision in the CTM, performed using a scalpel with a number 10 blade.^1^ Variation has been reported in the size of the CTM across population groups most of which is based on cadaver studies.^5^^,^^9^^,^^10^ CTM height has been reported as 7.5–10.5 mm in Western female cadavers, and much smaller, at 3.0–8.4 mm in the South Indian population.^9^^,^^10^ In addition, variation in CTM height according to age, sex, and height has been demonstrated in a recent study, which suggests having cricothyroidotomy sets of various outer diameters available.^4^, ^11^ Nutbeam and colleagues^12^ measured the height of the CTM on computed tomography scans of 482 trauma patients and demonstrated a statistically significant difference in CTM height between male and female patients, and noted that only 36% of the study population had a mean CTM height of 8.2 mm, suitable for the external diameter of a size 6.0 mm internal diameter tracheal tube. One of the limitations highlighted in this series was that because of the nature of trauma scans, these scans were performed with the neck in a neutral position. In contrast, the data from our study demonstrated that 80% of our study population had a CTM height of 8.2 mm in the neutral neck position. Although we did not consider racial identity while evaluating our results, we would like to acknowledge that the majority of our patients were White Irish females. A somewhat similar finding has been demonstrated in a recent study by Ansari and colleagues who studied the CTM height in a large subgroup of surgical patients in England, however, no mention of variation in CTM height based on ethnicity was made in that study.^8^ We consider this an important finding, considering this population subgroup would have been expected to have a greater CTM height compared with the South Asian population. It might be useful to evaluate the differences in height of CTM in more diverse populations in future.

The CTM is a dense fibroelastic trapezoidal membrane, bordered laterally by the cricothyroid muscles. It arises from the cricoid cartilage and stretches superiorly to the thyroid and arytenoid cartilages with the free superior margin being the vocal cord.^5^ Pliability of the CTM and movement at the cricothyroid joint may allow a larger tracheal tube to be passed than that predicted by measurement using ultrasound. However, placement of an oversized tracheal tube is known to result in fracture of the laryngeal cartilage in humans^13^ and traction on the borders of the CTM to open the space is unlikely to occur in the urgency of a critical event. Attempting to insert a tracheal tube with an outer diameter greater than the height of the CTM during cricothyroidotomy, may be associated with complications such as subglottic stenosis, laryngeal cartilage fracture, dysphonia, and hoarseness in the long term and perhaps failure to place a rescue airway in the first place.^5^ The American Association of Clinical Anatomists advise against using a cannula with an outer diameter larger than ∼8 mm during a cricothyroidotomy procedure.^14^ An *in vitro* comparative study of 21 cricothyroidotomy sets concluded that the outer diameter of many devices marketed for cricothyroidotomy are oversized for adult airway anatomy, particularly for females.^15^ Dixit and colleagues^16^ observed a 30% increase in CTM height with a change in neck position from neutral to maximal extension in a volunteer study with male and female participants. We were able to demonstrate only a 10% increase in CTM height with a change in neck position alone, but changes in CTM height of similar magnitude to Dixit and colleagues^16^ were observed only after insertion of an i-gel® in our study.

The i-gel® has been previously demonstrated to improve accuracy of identification of CTM by palpation in females.^17^ The obvious mechanism being the ventral displacement of the cricoid cartilage by the i-gel®® seated in the hypopharynx by virtue of cricoid cartilage being circumferential, unlike the tracheal rings which are incomplete posteriorly and more pliable. Cadaver dissections have demonstrated that the tip of the i-gel® by virtue of its small width and height, seats itself behind the cricoid cartilage in the cervical oesophagus. The wide middle section of the bowl is designed to abut the pharyngo-epiglottic folds, and the part distal to it becomes narrower and longer and creates an outward force against the tissues.^18^ This displacement of the cricoid cartilage with the i-gel® *in situ* is evident on ultrasound images, particularly when the head is extended.^17^ In the images obtained for the purpose of our study, there was evidence of what appeared to be stretching and thinning of the CTM on multiple images after i-gel® insertion (Fig. 2). The i-gel® also appeared to push the cricoid anteriorly as previously described. This study provides further evidence of the plasticity and flexibility of the CTM and surrounding structures, which is highly relevant to cricothyroidotomy.

The size 6 tracheal tube available for Plan D or eFONA (Difficult Airway Society guidelines for management of unanticipated difficult intubation in adults) in our institute is a Covidien Shiley™ Hi-Contour Oral/Nasal tracheal tube cuffed.^1^ We evaluated two other size 6 tracheal tubes available from the same provider. They differed in outer diameter, which would have an implication on ease of passage through the CTM. While the CTM height with i-gel® *in situ* and neck maximally extended was sufficient to allow passage of size 6 Hi-Contour and reinforced Shiley™ tracheal tubes in all patients, the size 6 Evac tube which is commonly used in intensive care units and has an outer diameter of 9.0 mm would be difficult to pass through the CTM in 2.5% of the patients, despite maximal neck extension and SAD in place (Table 3). It is important to note that in 20% of the patients, the CTM height would not be sufficient to accommodate the size 6 Evac tube despite maximal neck extension (with no SAD *in situ*). This finding highlights the importance of considering the outer diameter when selecting equipment to be available for emergency airway rescue via the CTM (Table 3).

**Table 3:** Proportion of study participants with measured CTM vertical measurement not sufficient to accommodate Covidien Shiley size 6 ET with different OD. Group A: neutral neck position, no SAD; Group B: extended neck position, no SAD; Group C: neutral neck position, SAD *in situ*; Group D: extended neck position, SAD *in situ*). CTM, cricothyroid membrane; ET, tracheal tube; SAD, supraglottic airway device.

Covidien Shiley^TM^ size 6 ET	Outer diameter (mm)	Group A, *n* (%)	Group B, *n* (%)	Group C, *n* (%)	Group D, *n* (%)
Hi-Contour	8.2	8 (20)	3 (7.5)	0 (0)	0 (0)
Reinforced	8.4	8 (20)	5 (12.5)	1 (2.5)	0 (0)
Evac with TaperGuard^TM^ cuff	9.0	9 (22.5)	8 (20)	3 (7.5)	1 (2.5)

### Strengths and limitations

This study demonstrates that the insertion of an i-gel® increases the height of the CTM. This finding could be clinically relevant given that this may lead to increased success during eFONA and decreased long-term complications related to forceful stretching of the membrane.

The limitations of our study were that we obtained four sequential images of CTM on each subject, which meant that we were unable to blind the principal investigator to the presence or absence of i-gel®. We did not measure the degree of neck extension in each patient but preferred to use ‘maximal’ neck extension. Although this may vary from patient to patient, it is the recommended position for cricothyroidotomy. We did not collect data on patient ethnicity; however, our patient population is predominantly of White Irish ethnicity. Given the differences demonstrated in CTM anatomy from previous studies it would have been interesting to evaluate women of different ethnicities in a subgroup analysis.

### Conclusions

Insertion of an i-gel® supraglottic airway device increases the vertical height of the cricothyroid membrane. Retaining or re-inserting an SAD may be beneficial during Emergency Front of Neck access.

## Author's contributions

Study design: AM, TD

Patient recruitment and data collection: AM, HMT

Measurements performed on images: SM

Statistical analysis: AM, TD

Drafting of manuscript: AM, SM, TD

Reviewing and editing the manuscript: all authors

## Funding source

This research did not receive any specific grant from funding agencies in the public, commercial, or not-for-profit sectors.

## Declarations of interest

The authors declare that they have no conflicts of interest.
